# Fluid Distribution Pattern in Adult-Onset Congenital, Idiopathic, and Secondary Normal-Pressure Hydrocephalus: Implications for Clinical Care

**DOI:** 10.3389/fneur.2017.00583

**Published:** 2017-11-01

**Authors:** Shigeki Yamada, Masatsune Ishikawa, Kazuo Yamamoto

**Affiliations:** ^1^Department of Neurosurgery, Normal Pressure Hydrocephalus Center, Rakuwakai Otowa Hospital, Kyoto, Japan; ^2^Department of Neurosurgery, Normal Pressure Hydrocephalus Center, Rakuwakai Otowa Hospital, Rakuwa Vila Ilios, Kyoto, Japan; ^3^Department of Neurosurgery, Rakuwakai Otowa Hospital, Kyoto, Japan

**Keywords:** normal-pressure hydrocephalus, cerebrospinal fluid, cerebrospinal fluid shunts, volumetric MRI, MRI volumetry

## Abstract

**Objective:**

In spite of growing evidence of idiopathic normal-pressure hydrocephalus (NPH), a viewpoint about clinical care for idiopathic NPH is still controversial. A continuous divergence of viewpoints might be due to confusing classifications of idiopathic and adult-onset congenital NPH. To elucidate the classification of NPH, we propose that adult-onset congenital NPH should be explicitly distinguished from idiopathic and secondary NPH.

**Methods:**

On the basis of conventional CT scan or MRI, idiopathic NPH was defined as narrow sulci at the high convexity in concurrent with enlargement of the ventricles, basal cistern and Sylvian fissure, whereas adult-onset congenital NPH was defined as huge ventricles without high-convexity tightness. We compared clinical characteristics and cerebrospinal fluid distribution among 85 patients diagnosed with idiopathic NPH, 17 patients with secondary NPH, and 7 patients with adult-onset congenital NPH. All patients underwent 3-T MRI examinations and tap-tests. The volumes of ventricles and subarachnoid spaces were measured using a 3D workstation based on T2-weighted 3D sequences.

**Results:**

The mean intracranial volume for the patients with adult-onset congenital NPH was almost 100 mL larger than the volumes for patients with idiopathic and secondary NPH. Compared with the patients with idiopathic or secondary NPH, patients with adult-onset congenital NPH exhibited larger ventricles but normal sized subarachnoid spaces. The mean volume ratio of the high-convexity subarachnoid space was significantly less in idiopathic NPH than in adult-onset congenital NPH, whereas the mean volume ratio of the basal cistern and Sylvian fissure in idiopathic NPH was >2 times larger than that in adult-onset congenital NPH. The symptoms of gait disturbance, cognitive impairment, and urinary incontinence in patients with adult-onset congenital NPH tended to progress more slowly compared to their progress in patients with idiopathic NPH.

**Conclusion:**

Cerebrospinal fluid distributions and disease progression were significantly different among the patients with adult-onset congenital NPH, idiopathic NPH and secondary NPH. This finding indicates that the pathogenesis of adult-onset congenital NPH may differ from those of idiopathic and secondary NPH. Therefore, adult-onset congenital NPH should be definitively distinguished from the categories of idiopathic and secondary NPH.

## Introduction

Since Adams and Hakim et al. reported patients diagnosed with normal-pressure hydrocephalus (NPH) in 1965 (1, 2), NPH has been categorized into idiopathic or secondary NPH developing after subarachnoid hemorrhage, trauma, infection, tumor, or with other conditions. We previously elucidated the differences of cerebrospinal fluid (CSF) distribution between idiopathic and secondary NPH (3). Typical CSF distribution in idiopathic NPH is that of a z-axial expansion of the ventricles in concurrence with enlargement of the basal cistern and Sylvian fissure and diminishment of the convexity subarachnoid spaces (4, 5). This characteristic CSF distribution is designated as disproportionately enlarged subarachnoid space hydrocephalus (DESH) (6). Conversely, typical CSF distribution in secondary NPH is the omnidirectional and cylindrical expansion of total ventricles in concurrence with severe diminishment of all parts of the subarachnoid spaces (3). However, we excluded the patients diagnosed with adult-onset congenital NPH from our previous volumetric analyses (3–5). Although case series with adult-onset congenital NPH have been reported (7–9), there has been no study to compare adult-onset congenital NPH with idiopathic or secondary NPH.

Recent clinical studies in robust diagnostic criteria for idiopathic NPH have shown the beneficial effects of shunting procedures for a patient with idiopathic NPH (10–17). However, some conservative experts strongly believe that shunt implantation is not effective for a patient with idiopathic NPH from their bitter experiences. We hypothesized that this divergence of viewpoints might be due to confusing classifications of idiopathic and adult-onset congenital NPH. Therefore, to elucidate the classification of NPH, we systematically compared the CSF distribution and clinical characteristics among the three categories of NPH, idiopathic, secondary, and adult-onset congenital NPH.

## Materials and Methods

### Study Population

**Figure 1:**
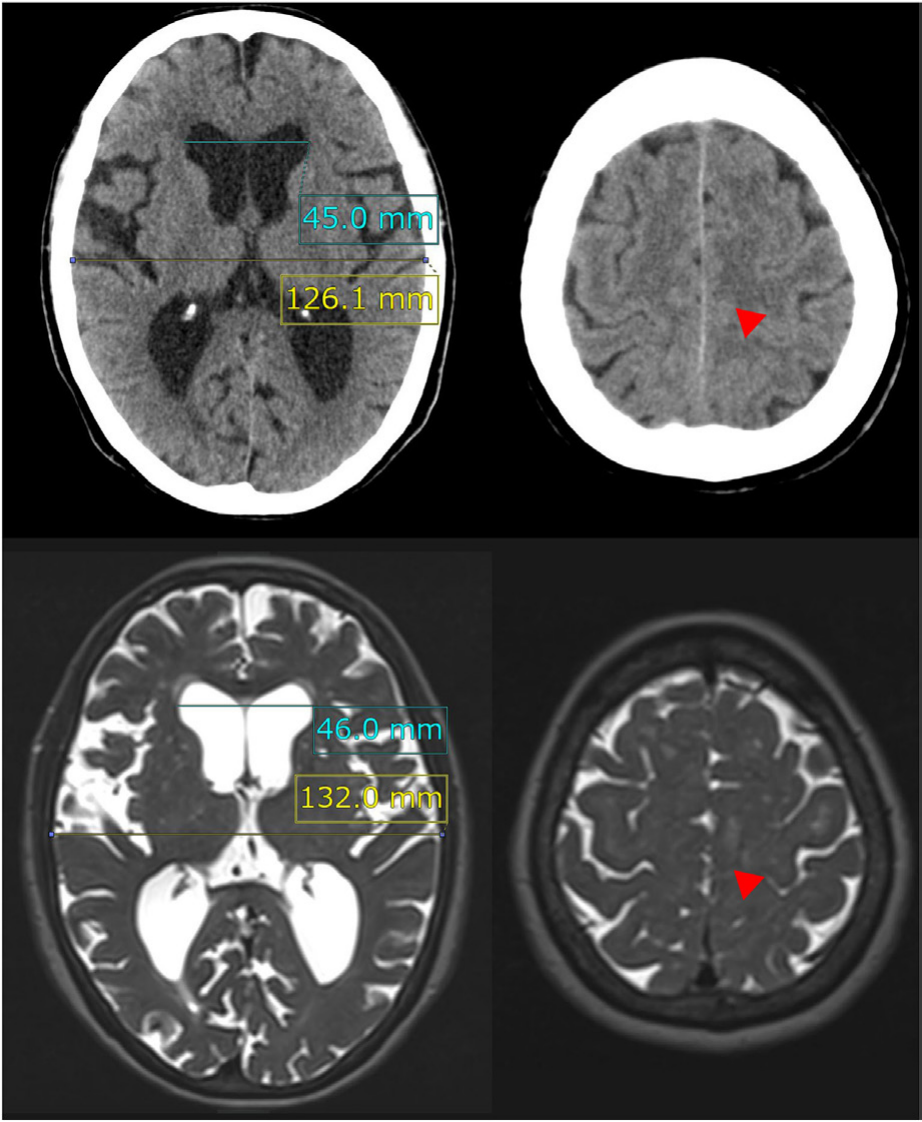
Evans index and high-convexity tightness. The upper figures show the traditional axial planes on CT scan and the lower figures show the T2-weighted MRI in a representative case of idiopathic NPH. The Evans index is measured as the maximal width of the frontal horns of the bilateral ventricles (sky blue line) to the maximal width of the internal diameter of the cranium (yellow line) on the basis of the axial planes on CT or MRI. In this case, the Evans index was calculated as 0.36 on CT scan and 0.35 on MRI. Red arrowheads indicate the high-convexity tightness which means decreased subarachnoid spaces at the convexity.

The study design and protocol were approved by the ethics committee for human research at our institute. Since we began to evaluate CSF distribution using T2-weighted 3D sampling perfection with the application of optimized contrast using the variable flip-angle evolution (SPACE) sequences in November 2013, and prospectively collected clinical data and conducted MRI examinations. Details of the clinical data collection, image acquisition, and segmentation and quantification of the ventricular and subarachnoid space are described in our previous publications (3–5). In brief, 130 patients underwent a CSF tap-test and MRI examination for CSF volume analysis after providing written informed consent. The CSF tap-test involves the removal of 30–40 mL of CSF via a lumbar tap. Responses to the CSF tap-test were assessed using a Japanese idiopathic NPH grading scale, and by quantitative examination of gait and cognition before and one day and four days after the CSF tap-test. Gait was quantitatively assessed using the 3-m timed up and go test (TUG) and 10-m straight walking test (SWT). Cognition was quantitatively assessed using the mini-mental state examination (MMSE) and frontal assessment battery (FAB). Positive response to the CSF tap-test was defined as ≥10% improvement of the best changes in any of the quantitative examinations or ≥1 point of improvement of the Japanese idiopathic NPH grading scale. Idiopathic NPH was defined as DESH appearance, especially high-convexity tightness on the conventional CT scan or MRI (Figure 1) and any symptoms of gait disturbance, cognitive dysfunction, and incontinence without any causative episodes of hydrocephalus. Additionally, all 85 patients diagnosed with idiopathic NPH had responses to the CSF tap-test. Adult-onset congenital NPH was defined as huge ventricles without DESH fashion or high-convexity tightness on the conventional CT scan or MRI and any triad symptoms same as idiopathic NPH. Seven patients were diagnosed with adult-onset congenital NPH, regardless of responses to the CSF tap-test. Secondary NPH was defined as panventriculomegaly and progressive symptoms of impaired consciousness and/or gait disturbance subsequent to subarachnoid or intracerebral hemorrhage or traumatic brain injury. Eight of 85 patients with idiopathic NPH and 11 of 17 patients with secondary NPH could not be assessed any quantitative examinations, because they could not stand and answer any questions.

### Image Acquisition and Volumetric Analysis

All MRI examinations were performed with a 64-channel 3-T MRI system (MAGNETOM Skyra, Siemens AG, Munich, Germany). Volumetric data were delivered to a 3D workstation (SYNAPSE 3D; Fujifilm Medical Systems, Tokyo, Japan), and semi-automatically segmented into intracranial space, ventricles, and subarachnoid spaces. The subarachnoid spaces were segmented into the following three components: convexity subarachnoid space, Sylvian fissure and basal cistern and posterior fossa. Both, the mean volumes and volume ratios (in percentages), which were calculated as the measured volumes divided by the intracranial volume, were analyzed to eliminate the effect of head size.

### Morphological Indices Specific to NPH

**Figure 2:**
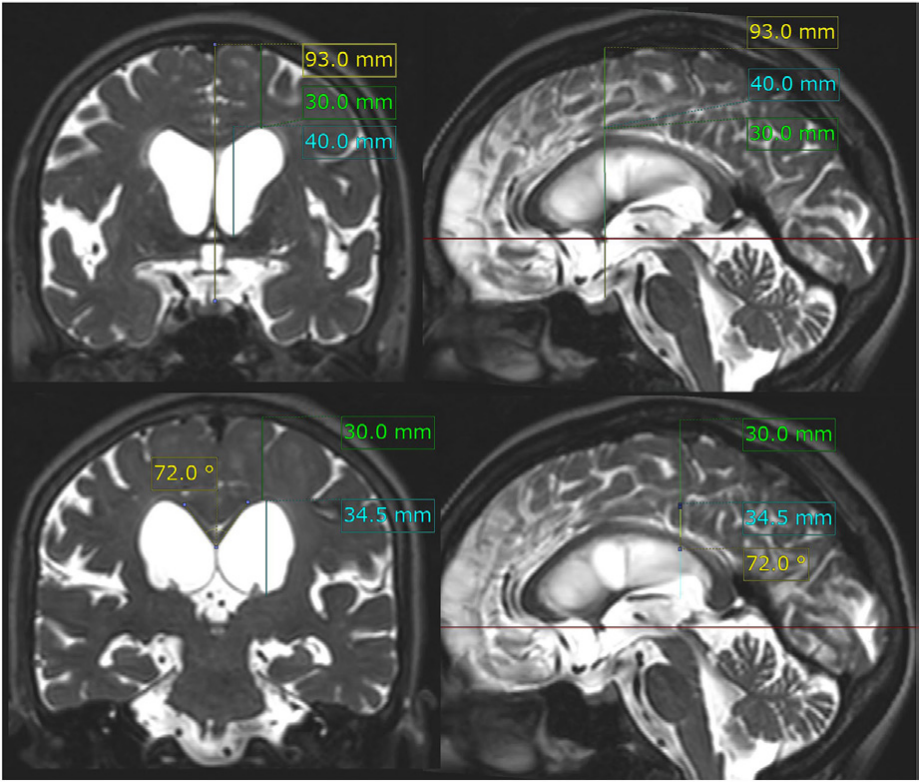
z-Evans index, brain per ventricle ratios (BVRs) at the anterior commissure (AC) and posterior commissure (PC) levels, and callosal angle. The z-Evans index is measured as the maximum z-axial length of the frontal horns of the lateral ventricles (sky blue line) to the maximum cranial z-axial length at the midline (yellow line) on the coronal plane (left upper) just on the AC (right upper). The BVRs at the AC and PC levels are measured as the maximum z-axial length of the brain just above the lateral ventricles (green line) divided by the maximum length of the lateral ventricles (sky blue line). The callosal angle is measured as the roof of the bilateral ventricles on the coronal plane (left lower) just on the PC (right lower). In this case same as Figure 1, the z-Evans index, BVRs at the AC and PC levels, and callosal angle were calculated as 0.43, 0.75, 0.87, and 72°, respectively.

The Evans index was measured as the maximal width of the frontal horns of the lateral ventricles to the maximal width of the internal diameter of the cranium based on the X dimension, as shown in Figure 1. The z-Evans index (Figure 2) was measured as the maximum z-axial length of the frontal horns of the lateral ventricles to the maximum cranial z-axial length on the coronal plane, which was perpendicular to the anteroposterior commissure (PC) plane on the anterior commissure (AC) (4). The callosal angle (Figure 2) was measured as the angle of the roof of the bilateral ventricles on the coronal plane at the PC level (18). The brain per ventricle ratios (BVRs) at the AC and PC (Figure 2) were calculated as the maximum width of the brain just above the lateral ventricles divided by the maximum width of the lateral ventricles on the reference coronal planes at the AC and PC levels, respectively (5). The severities of periventricular and deep white matter hyperintensities were evaluated using the Fazekas rating scale on the fluid attenuated inversion recovery sequence (19).

### Statistical Analysis

Mean values and SDs for age and other characteristics and indices were calculated in the three NPH groups. These parameters and CSF volumes in the patients with adult-onset congenital NPH were compared with those in the patients with idiopathic NPH or secondary NPH using the Mann–Whitney–Wilcoxon test, because the majority of them did not have normal distributions in the Shapiro–Wilk test. Statistical significance was indicated by *P* < 0.05. Missing data were treated as deficit data, and therefore did not affect other variables. Statistical analyses were performed using the R software (version 3.3.2; R Foundation for Statistical Computing, Vienna, Austria; http://www.R-project.org).

## Results

### Clinical Characteristics

Of the 130 patients who underwent CSF tap-tests and MRI examinations in our NPH center, 85 patients were diagnosed with idiopathic NPH, 17 with secondary NPH, and 7 with adult-onset congenital NPH. The other 21 patients who were diagnosed with Alzheimer disease or Parkinson disease and without any types of NPH were excluded from this study. Table 1 presents the clinical characteristics and morphological indices for the three NPH categories. The range of the duration from initial presentation of symptoms until CSF tap-tests and MRI examinations was from 1 month to 7 years (mean, 2.3 years) among the patients with idiopathic NPH, whereas it was 1–10 years (mean; 5.7 years) for the patients with adult-onset congenital NPH. Additionally, the duration from the diagnosis of NPH to receiving shunt surgery for the patients with adult-onset congenital NPH was the longest among the three NPH categories, which indicate that the symptoms in the patients with adult-onset congenital NPH tended to be mild and slowly progressive compared with those of idiopathic and secondary NPH. The mean values of the Evans and z-Evans indices for the patients with adult-onset congenital NPH were significantly greater than for those with idiopathic and secondary NPH, whereas the callosal angle was not significantly different.

**Table 1:** Clinical characteristics of the study population.

	Idiopathic NPH	Adult-onset congenital NPH	Secondary NPH
Total number (man/woman)	85 (52/33)	7 (4/3)	17 (10/7)
Age, years	76.9 ± 6.2	73.3 ± 5.6	72.7 ± 10.2
Disease duration,^a^ months	27.8 ± 20.3*	68.6 ± 43.1	7.2 ± 12.4*
modified Rankin scale	2.77 ± 0.91	2.57 ± 0.79	3.94 ± 1.09*
Gait domain on iNPHGS	2.54 ± 0.66	2.71 ± 0.76	3.47 ± 1.01*
Cognitive domain on iNPHGS	1.98 ± 0.98	1.86 ± 0.90	3.53 ± 0.94*
Urinary domain on iNPHGS	1.82 ± 1.13	1.57 ± 1.13	3.41 ± 1.12*
Evans index	0.34 ± 0.04*	0.40 ± 0.05	0.34 ± 0.04*
Z-Evans index	0.44 ± 0.06*	0.58 ± 0.14	0.39 ± 0.04*
BVR at AC	0.73 ± 0.18*	0.50 ± 0.13	0.93 ± 0.19*
BVR at PC	0.92 ± 0.25*	0.58 ± 0.17	1.31 ± 0.39*
Callosal angle, degree	63.7 ± 17.8	72.7 ± 29.7	82.3 ± 17.0

*iNPHGS, iNPH grading scale before CSF tap-test; BVR, brain per ventricle ratio; AC, anterior commissure; PC, posterior commissure*.

*^a^Disease duration; time interval from the initial symptom onset until MRI examination*.

*Statistically significant difference from the congenital NPH value (*P* < 0.05).

### Comparison of CSF Distribution

**Figure 3:**
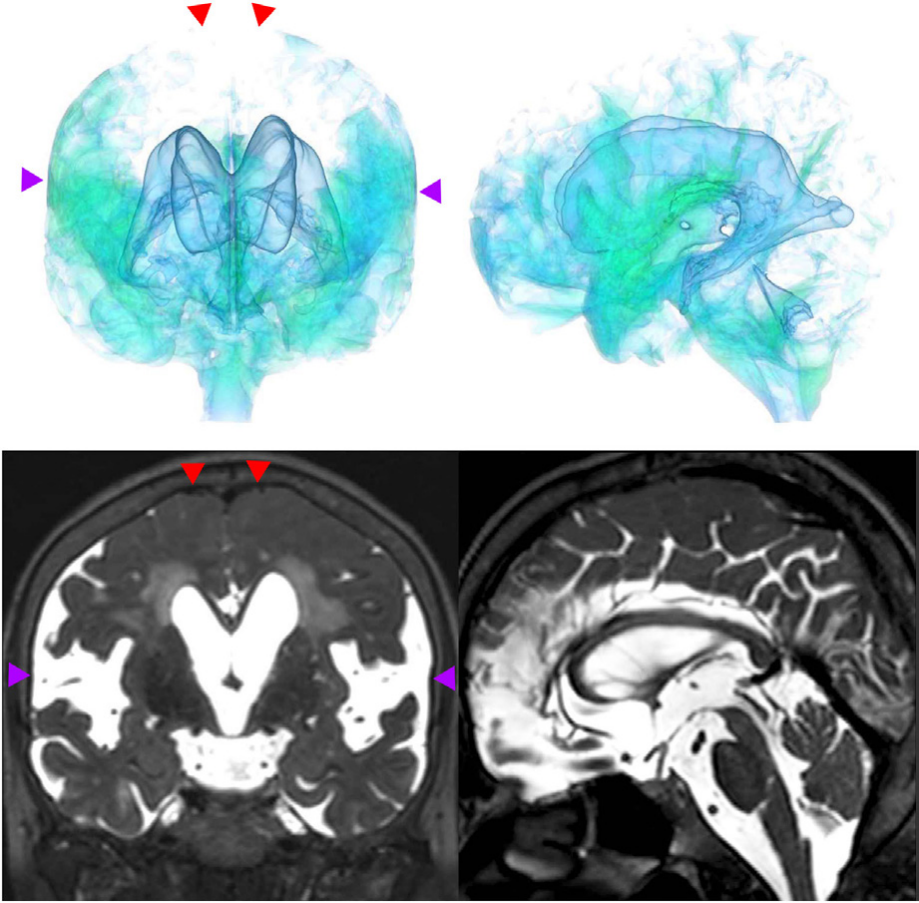
A representative case of idiopathic NPH. The bilateral ventricles expanded toward the z-axial vertex and the Sylvian fissure enlarged (purple arrowhead) and the sulci at the high convexity reduced (red arrowhead).

**Figure 4:**
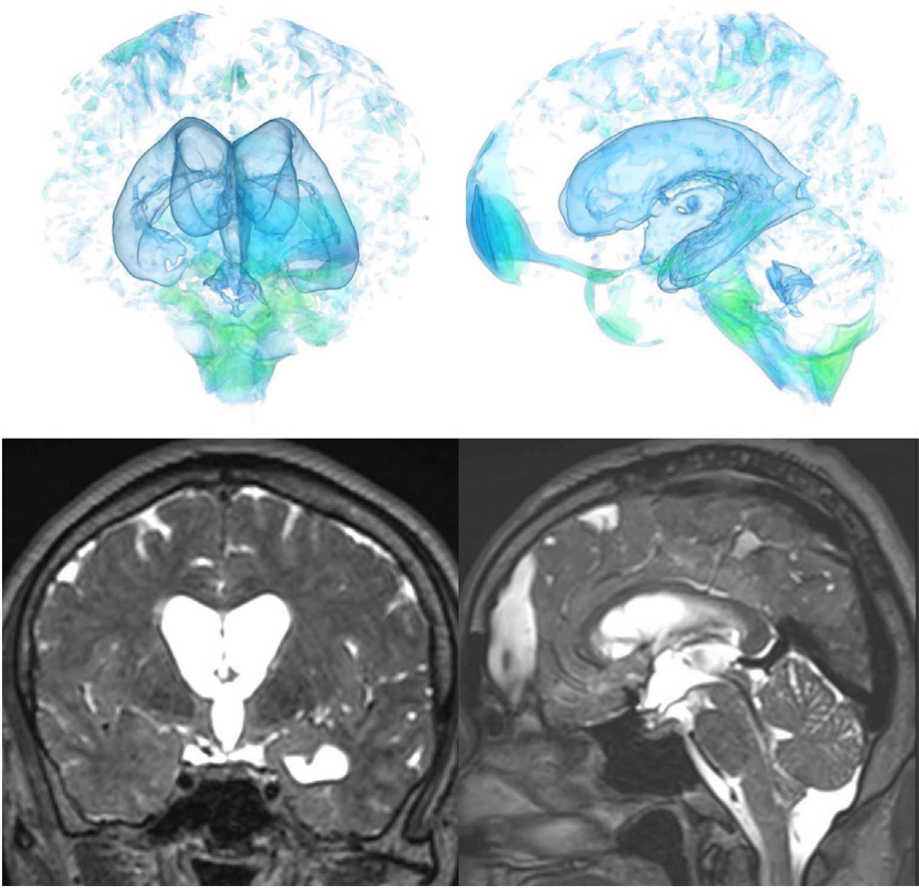
A representative case of secondary NPH. The bilateral ventricles expanded symmetrically in all directions and the Sylvian fissure reduced and the sulci at the high convexity were visible.

**Figure 5:**
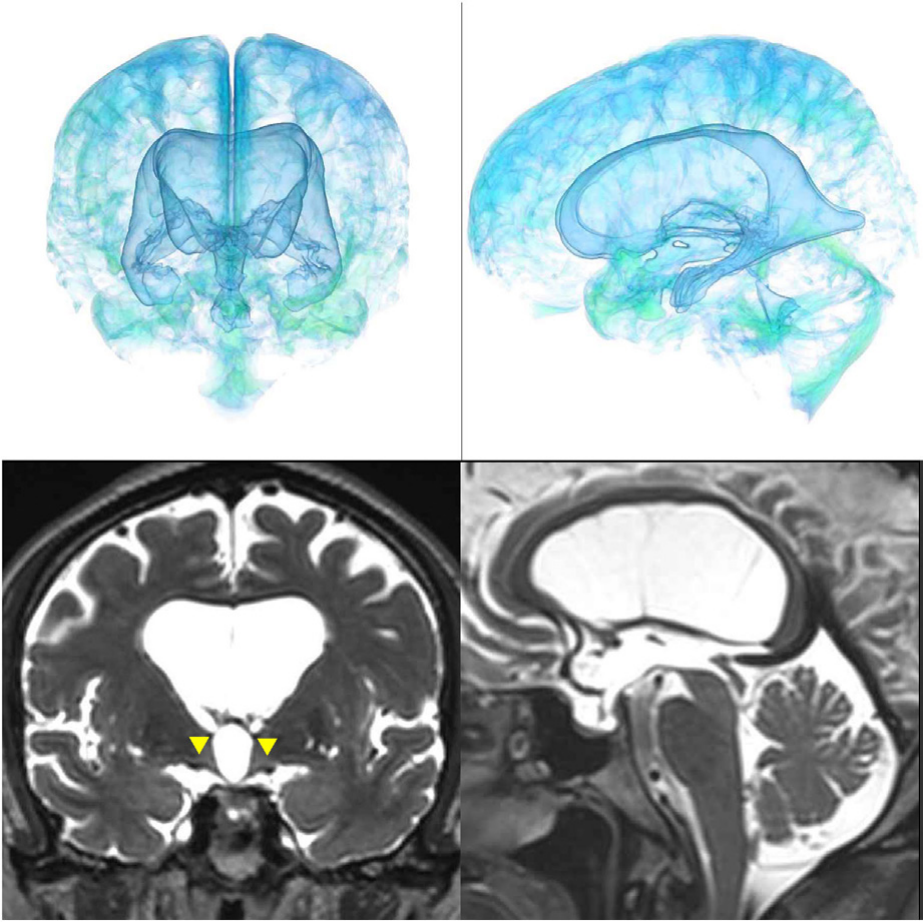
Case 1 diagnosed with adult-onset congenital NPH. A man aged 70 years old had huge lateral ventricles due to severe stenosis at the bilateral foramens of Monro (yellow arrowhead).

**Figure 6:**
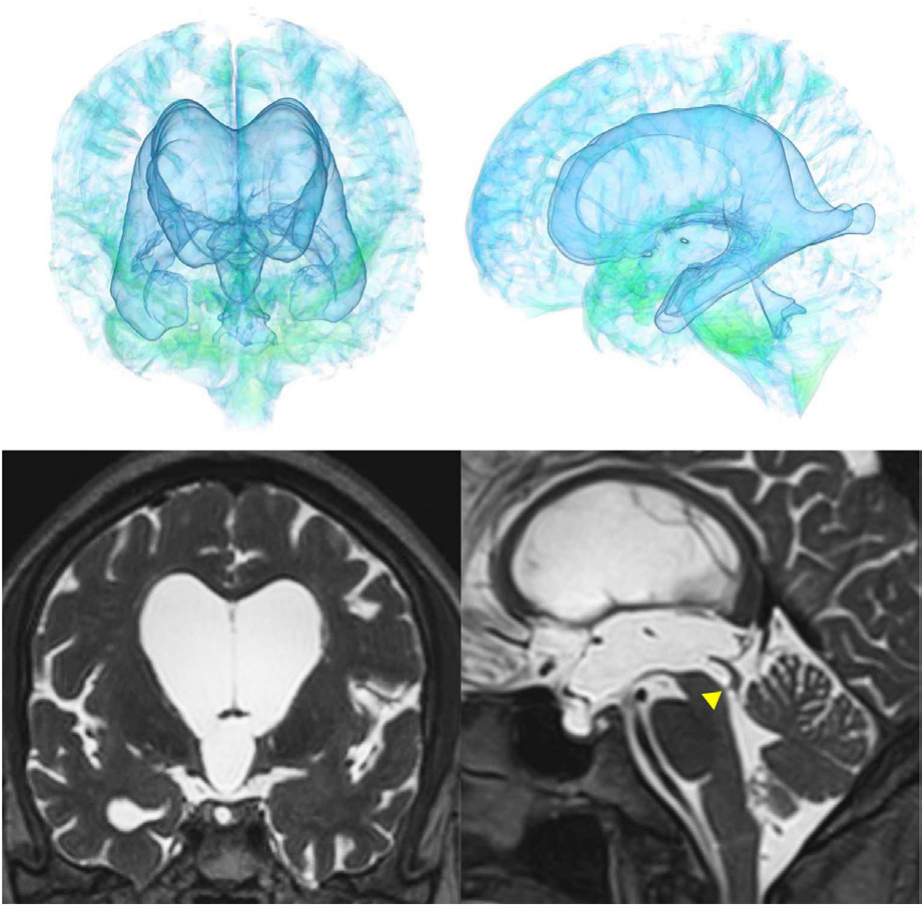
Case 2 diagnosed with adult-onset congenital NPH. A man aged 75 years old had huge lateral ventricles due to obstruction of the cerebral aqueduct by a membrane (yellow arrowhead) on sagittal thin-slice MRI.

**Figure 7:**
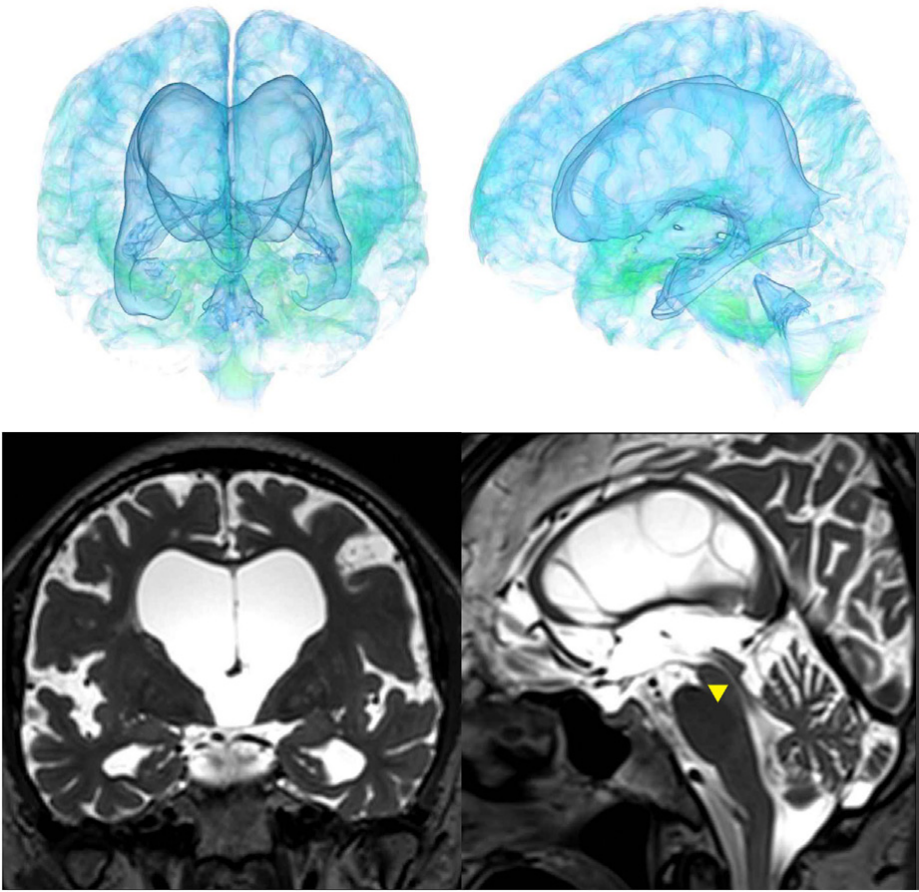
Case 3 diagnosed with adult-onset congenital NPH.

**Figure 8:**
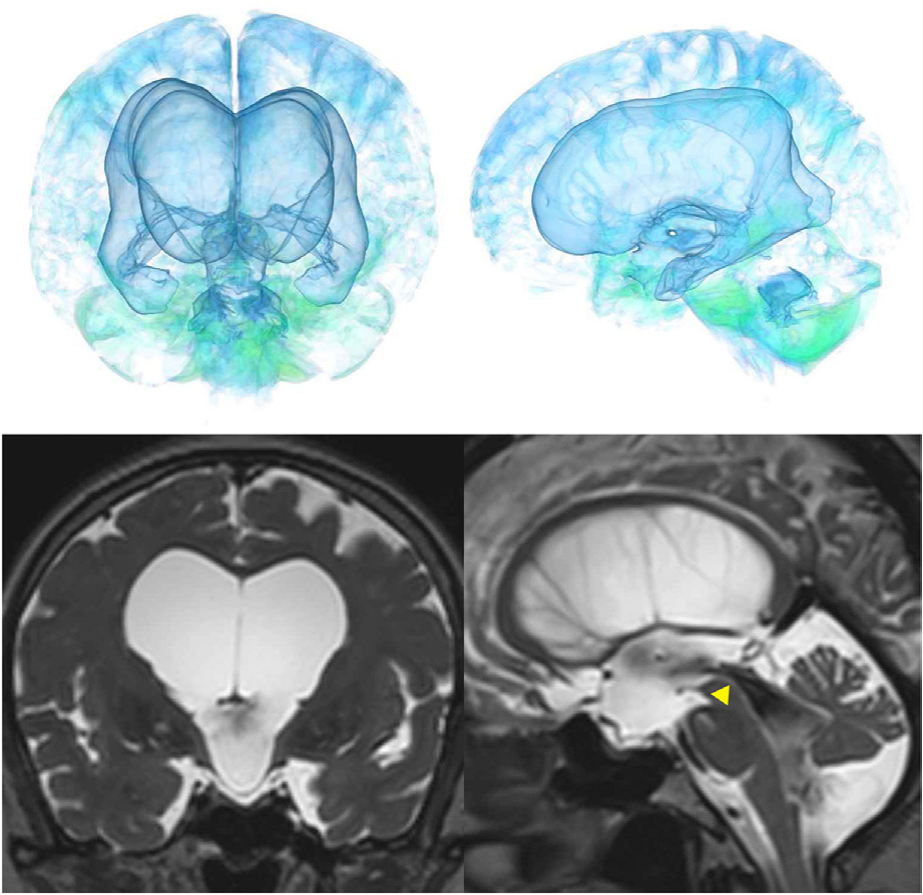
Case 4 diagnosed with adult-onset congenital NPH.

**Figure 9:**
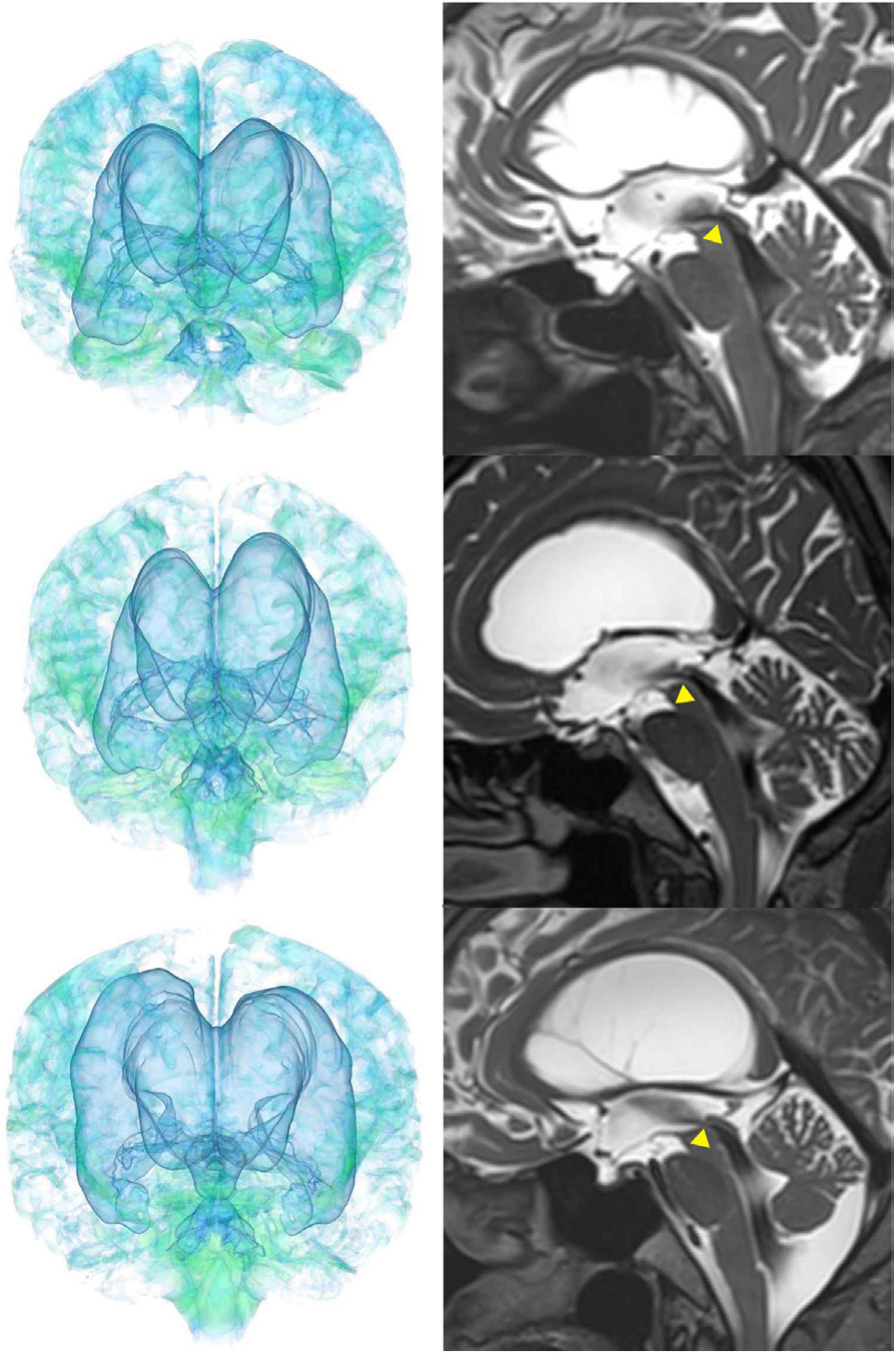
Three patients diagnosed with adult-onset congenital NPH. There was no evidence of obstructions to CSF flow in the ventricles. The sagittal planes show jet flow voids in the third ventricle from the cerebral aqueduct (yellow arrowheads).

The patients with idiopathic NPH showed lateral ventricles expanded to the vertex, enlarged Sylvian fissure and severely diminished convexity subarachnoid spaces (Figure 3), whereas in the patients with secondary NPH, all of the ventricles were expanded symmetrically in all directions and severe diminishment of the subarachnoid spaces was observed (Figure 4). The seven patients with adult-onset congenital NPH had variously shaped large ventricles, although the volume and distribution of the subarachnoid spaces were within the normal range (Figures 5–9). Of the seven patients with adult-onset congenital NPH, one showed severe stenosis at the bilateral foramen of Monro (Figure 5), and one an obstruction of the cerebral aqueduct by a membrane (Figure 6). The other five patients exhibited enlargement of all ventricles, without a downward bulging third ventricular floor (Figures 7–9). These five patients met the diagnostic criteria for panventriculomegaly with a wide foramen of Magendie and a large cisterna magna, which was recently described by Kageyama et al. (9).

The mean intracranial volume for the patients with adult-onset congenital NPH was 1,621 ± 128 mL, which was 100 mL larger than the volumes for patients with idiopathic NPH (1,516 ± 150 mL) and secondary NPH (1,474 ± 140 mL), as presented in Table 2. The mean volumes and volume ratios of the total intracranial CSF spaces and total ventricles in the patients with adult-onset congenital NPH were significantly larger than for those in the patients with idiopathic and secondary NPH. Although the total and posterior fossa of subarachnoid spaces in the patients with idiopathic and congenital NPH had similar mean volumes, the mean volumes of the convexity subarachnoid space and the basal cistern and Sylvian fissure were reversed. Interestingly, the convexity subarachnoid space in the patients with congenital NPH was significantly larger than those in the patients with idiopathic and secondary NPH (Table 2), although the BVRs at the AC and PC in the patients with congenital NPH were significantly smaller than those in the patients with idiopathic and secondary NPH (Table 1). The cortical thickness of adult-onset congenital NPH is thin, but there is no finding of brain compression like as idiopathic NPH.

**Table 2:** Mean volume (mL) and ratio (%) of brain parenchyma and CSF.

	Idiopathic NPH (85)	Adult-onset congenital NPH (7)	Secondary NPH (17)
Intracranial volume	1,516 ± 150	1,621 ± 128	1,474 ± 140*
Brain parenchyma	1,093 (72.1%)*	1,092 (67.3%)	1,176 (80.0%)*
Total CSF	423* (27.9%)*	529 (32.7%)	297* (20.1%)*
Total ventricles	162* (10.7%)*	275 (17.0%)	121* (8.2%)*
Bilateral ventricles	150* (9.8%)*	259 (16.0%)	112* (7.6%)*
Third ventricle	5.1* (0.3%)*	10.1 (0.6%)	5.2* (0.3%)*
Fourth ventricle	3.7 (0.2%)	5.5 (0.3%)	4.4 (0.3%)
Total subarachnoid spaces	262 (17.2%)	254 (15.7%)	176* (11.9%)
Convexity subarachnoid space	75.8* (5.0%)*	128 (7.9%)	75.7* (5.1%)*
Basal cistern and Sylvian fissure	122* (8.0%)*	62.0 (3.8%)	54.8 (3.7%)
Posterior fossa	64.4 (4.2%)	63.7 (3.9%)	45.5* (3.1%)*

*Statistically significant difference from the adult-onset congenital NPH value (*P* < 0.05).

The mean times on TUG and SWT before and after the CSF tap-test in the patients with idiopathic NPH were longer than those in the patients with adult-onset congenital NPH, although there was no statistical difference, because of large SDs (Table 3). The mean scores and their changes of MMSE and FAB before and after the CSF tap-test in the patients with idiopathic NPH were approximately equal to those in the patients with adult-onset congenital NPH.

**Table 3:** CSF pressure and change of measurements for gait and cognition at CSF tap-test.

	Idiopathic NPH (77)	Adult-onset congenital NPH (7)	Secondary NPH (6)
Age, years	77.1 ± 5.9	73.3 ± 5.6	71.8 ± 5.1
CSF pressure, cmH_2_O	13.1 ± 2.4	13.7 ± 2.3	10.0 ± 3.0
TUG time before tap, s	36.1 ± 55.3	19.6 ± 14.8	13.7 ± 4.3
TUG time after tap, s	29.6 ± 38.5	16.7 ± 13.7	13.2 ± 7.1
Δ TUG time at tap, s	8.4 ± 31.1	2.2 ± 2.6	0.5 ± 6.1
SWT time before tap, s	22.4 ± 25.5	13.8 ± 6.8	10.1 ± 3.6
SWT time after tap, s	21.1 ± 25.4	11.6 ± 5.8	10.5 ± 3.3
Δ SWT time at tap, s	3.2 ± 19.1	1.5 ± 1.7	-0.3 ± 2.0
180° turn before tap, step	8.5 ± 7.4	5.4 ± 3.1	4.8 ± 1.5
180° turn after tap, step	8.1 ± 8.1	4.2 ± 2.6	3.5 ± 1.3
Δ 180° turn at tap, step	1.1 ± 4.9	0.2 ± 1.6	1.3 ± 1.7
MMSE before tap, score	21.9 ± 6.0	22.9 ± 2.9	16.5 ± 8.3
MMSE after tap, score	23.8 ± 5.8	26.0 ± 3.3	20.3 ± 6.2
Δ MMSE at tap, score	2.0 ± 2.8	3.1 ± 1.7	3.8 ± 2.5
FAB before tap, score	10.0 ± 3.1	11.4 ± 2.0	7.5 ± 5.1
FAB after tap, score	11.3 ± 3.2	11.9 ± 2.3	9.8 ± 1.7
Δ FAB at tap, score	1.3 ± 2.3	0.4 ± 1.8	2.3 ± 3.9

*Δ; difference between times, steps or scores before and after the tap-test*.

*TUG, timed 3-m up & go test; SWT, 10-m straight walking test; MMSE, mini-mental state examination; FAB, frontal assessment battery*.

## Discussion

The morphological characteristics of intracranial CSF spaces in adult-onset congenital NPH are clearly distinguishable from those in idiopathic and secondary NPH. We summarize the clinical and radiological characteristics for the three NPH categories in Table 4. The key feature for the diagnosis of idiopathic NPH is compression of the convexity part of the brain in concurrent with decreased subarachnoid spaces at the convexity due to z-axial expansion of bilateral ventricles and Sylvian fissure, namely, “DESH” (4–6, 20, 21). Particularly, panventriculomegaly is not necessary for the current diagnosis of idiopathic NPH. In contrast, the patients with adult-onset congenital NPH showed variously shaped huge ventricles and thin brain, but their subarachnoid spaces were within the normal size and distribution. Additionally, the mean intracranial volume for the patients with adult-onset congenital NPH was greater than that for those with idiopathic and secondary NPH. Our cases of adult-onset congenital NPH resemble the cases subcategorized as idiopathic NPH with a large head circumference or intracranial volume as previously reported (8, 22, 23). Graff-Radford and Godersky also reported that around 10% of the patients presenting clinically with NPH have congenital chronic NPH, which may turn symptomatic at an older age, as evidenced by the fact that they have a head size above the 98th percentile (24).

**Table 4:** Representative characteristics of each type of NPH.

	Idiopathic NPH	Adult-onset congenital NPH	Secondary NPH
Age	65–90 years	40–90 years	40–90 years
Characteristics of symptoms	Disturbance of gait, cognition and continence	Disturbance of gait, cognition and continence	Impaired consciousness and gait disturbance
Progress of symptoms	1 month to 7 years	1–10 years	Less than 6 months
Combined anomalies	None	Aqueductal stenosis, posterior fossa anomaly (ex. Blake’s pouch cyst), LOVA and PaVM	None
Preceding disorders	None	Genetic factors, ex. dysfunction of motile cilia	Subarachnoid hemorrhage, meningitis, brain injury
Concurrent disease	Alzheimer disease, spinal stenosis, and spondylosis. etc.	None	None
Neuroimaging feature	DESH	Panventriculomegaly without DESH fashion	Panventriculomegaly with close cistern and without DESH fashion
Lateral ventricle	Z-directional expansion	Huge and various shape	Cylindrical expansion
Third ventricle	Not enlarged	Enlarged widely	Enlarged widely
BVR	Narrow (<1.0 at AC, <1.5 at PC)	Severely narrow (<0.6 at AC, <0.75 at PC)	Not narrow (>0.6 at AC, >0.75 at PC)
Callosal angle	Sharp (30–110°)	Wide range (45–135°)	Not sharp (70–100°)

*LOVA, long-standing overt ventriculomegaly in adults; PaVM, panventriculomegaly with a wide foramen of Magendie and a large cisterna magna; DESH, disproportionately enlarged subarachnoid space hydrocephalus; AC, anterior commissure; PC, posterior commissure; BVR, brain per ventricle ratio*.

The Japanese guideline for management of idiopathic NPH classifies congenital/developmental etiologies of NPH into secondary NPH subcategory in order to exclude it from the idiopathic NPH category (20). The international idiopathic NPH guideline treats aqueductal stenosis, long-standing overt ventriculomegaly syndrome, and non-communicating hydrocephalus as other hydrocephalus disorders, and clearly exclude them from the category of idiopathic NPH (25). Although there are numerus articles reporting good outcomes of shunt surgery for idiopathic NPH (10–17), many neurosurgeons and neurologists still have the negative impression that idiopathic NPH is difficult to treat with a shunt surgery (26, 27). This divergence of opinions might be due to confusing classifications of idiopathic and adult-onset congenital NPH. The ancient definition of idiopathic NPH as ventriculomegaly without any preceding brain diseases often miscombines adult-onset congenital NPH with idiopathic NPH. The surgical indication and time of recommending shunt surgery are also different between congenital and idiopathic NPH, because the symptoms in patients with adult-onset congenital NPH tend to progress more slowly and improve to a lesser extent after shunt surgery, compared to those in patients with idiopathic NPH (28, 29). Therefore, adult-onset congenital NPH should be clearly distinguished from the categories of idiopathic and secondary NPH. Definitive distinction of adult-onset congenital NPH would enable the estimation of accurate prevalence rates and prediction of clinical outcomes following shunt surgery in each category of NPH.

The present study has several limitations. The classifications of idiopathic and adult-onset congenital NPH were only based on the brain imaging, because their symptoms resembled closely. Therefore, it was the logical result that CSF distribution pattern in adult-onset congenital NPH differed from that in idiopathic NPH. The diagnosis of adult-onset congenital NPH did not include confirmation of the congenital etiology, either by genetic analyses or by evidence of ventriculomegaly during childhood, although one patient with congenital NPH had a family history of NPH. Recent developments in genetic studies have facilitated detection of susceptibility genes for many late-onset genetic disorders. For example, panventriculomegaly with a wide foramen of Magendie and a large cisterna magna is associated with a DNAH14 mutation that contributes to motile cilia (9). Additionally, there were few patients with adult-onset congenital NPH. However, the prevalence of adult-onset congenital NPH is estimated at around 10% compared with the incidence of idiopathic NPH (24). In elderly populations, idiopathic NPH is known to be the most common type of NPH (24, 30–33). Therefore, seven patients with adult-onset congenital NPH and 85 patients with idiopathic NPH in our case series were represented the realistic frequency. However, our findings should be further assessed in larger populations of patients with each category of NPH. Finally, we cannot appropriately explain the progressive nature of the symptoms of patients with adult-onset congenital NPH despite the lack of evidence of brain compression. Further study is warranted to elucidate the mechanisms underlying the progression of symptoms in the patients with adult-onset congenital NPH and idiopathic NPH. And someday in the future, we would change the name of “idiopathic” adequately.

## Ethics Statement

Full name of the ethics committee is “Ethics committee of Rakuwakai Otowa Hospital.” The study design and protocol were approved by the ethics committee for human research at our institute. Authorization number is RAKUOTO-RIN-14-003. All participants in this study were provided written informed consent.

## Author’s Note

Dr. Shigeki Yamada MD, PhD learned biostatistics at the Department of the Health and Environmental Sciences, Kyoto University School of Public Health from 2001 to 2004.

## Author Contributions

SY had substantial contributions to the conception or design of the work, acquisition of data, statistical analysis, and interpretation of data. Statistical analysis conducted by SY. MI had substantial contributions to the study concept and design, critical revision of manuscript for intellectual content, and supervision. KY had substantial contributions to the acquisition of data and study supervision.

## Conflict of Interest Statement

SY received speaker’s honoraria from Johnson & Johnson, Nihon Medi-Physics, and Fujifilm Medical Systems. Masatsune Ishikawa received grant funding from Japan’s Ministry of Health and Welfare, as well as honoraria from Johnson and Johnson, Nihon-Medi-Physics and Medtronic Japan Co., Ltd. (Japan) for speaking at seminars. MI received grant funding from Japan’s Ministry of Health and Welfare, as well as honoraria from Johnson and Johnson, Nihon-Medi-Physics and Medtronic Japan Co., Ltd. (Japan) for speaking at seminars. KY declares no disclosures or conflicts of interest.
